# Establishment of the African Medicines Agency: progress, challenges and regulatory readiness

**DOI:** 10.1186/s40545-020-00281-9

**Published:** 2021-03-08

**Authors:** Bakani Mark Ncube, Admire Dube, Kim Ward

**Affiliations:** Department of Pharmacy Practice, School of Pharmacy, University of the Western Cape, Private Bag X17, Bellville, 7535 South Africa

**Keywords:** Pharmaceutical policy, Medicines regulatory harmonisation, African Medicines Regulatory Harmonisation Initiative, AU model law on medical products regulation, African Medicines Agency

## Abstract

Insufficient access to quality, safe, efficacious and affordable medical products in Africa has posed a significant challenge to public health for decades. In part, this is attributed to weak or absent policies and regulatory systems, a lack of competent regulatory professionals in National Medicines Regulatory Authorities (NMRAs) and ineffective regional collaborations among NMRAs. In response to national regulatory challenges in Africa, a number of regional harmonisation efforts were introduced through the African Medicines Regulatory Harmonisation (AMRH) initiative to, among others, expedite market authorisation of medical products and to facilitate the alignment of national legislative frameworks with the AU Model Law on Medical Products Regulation. The goals of the model law include to increase collaboration across countries and to facilitate the overall regional harmonisation process. The AMRH initiative is proposed to serve as the foundation for the establishment of the African Medicines Agency (AMA). The AMA will, as one of its mandates, coordinate the regional harmonisation systems that are enabled by AU Model Law domestication and implementation. In this paper, we review the key entities involved in regional and continental harmonisation of medicines regulation, the milestones achieved in establishing the AMA as well as the implementation targets and anticipated challenges related to the AU Model Law domestication and the AMA’s establishment. This review shows that implementation targets for the AU Model Law have not been fully met, and the AMA treaty has not been ratified by the minimum required number of countries for its establishment. In spite of the challenges, the AU Model Law and the AMA hold promise to address gaps and inconsistencies in national regulatory legislation as well as to ensure effective medicines regulation by galvanising technical support, regulatory expertise and resources at a continental level. Furthermore, this review provides recommendations for future research.

## Introduction

Africa is a continent with 55 countries and a population of about 1.2 billion people [1, 2]. The continent also has a high burden of communicable and non-communicable diseases which presents significant challenges for the health care system [1]. For instance, the World Health Organization (WHO) African Regional Office (AfRO) reports having 11% of the world’s population yet it bears a disproportionate burden of disease with 60% of people living with HIV/AIDS and more than 90% of the annual global malaria cases being in Africa [3]. Insufficient access to quality, safe, efficacious and affordable medical products in Africa has posed a significant challenge to public health for decades [4, 5]. Amongst low- and middle-income countries worldwide, the African region has the highest prevalence of poor-quality medicines with an 18.7% prevalence of substandard and falsified medicines [6]. These challenges have largely been attributed to weak or absent medicines regulatory systems [4, 7], which include unclear policies, as well as incomplete or incoherent legal and regulatory frameworks. In addition, challenges with high staff turnover and lack of competent regulatory professionals in National Medicines Regulatory Authorities (NMRAs) together with poor regulatory infrastructure and ineffective regional collaborations among NMRAs exist [4, 5, 7–19]. All countries in Africa, with the exception of Sahrawi Republic, have an NMRA or an administrative unit conducting some or all expected NMRA functions [20]. However, the level of regulatory oversight on the continent has wide divergence with some countries having robust and functional NMRAs, whereas other countries have regulatory systems that are virtually non-existent [5, 7, 13–15, 20, 21].

Due to regulatory legislation being created at the national level, neighbouring countries within Regional Economic Communities (RECs) can have considerably different procedures and systems for regulating as well as approving medical products [4, 14, 15]. Consequently, in cases where the NMRAs receive evidence dossiers that are identical, countries are under no obligation to adopt the regulatory decisions that have been made in another country [4, 15]. Applicants and manufacturers are obligated to submit duplicative evidence dossiers to several NMRAs for the registration of medical products in each country where the product is intended for marketing [4, 14, 15, 22]. Each submission of a dossier has time and cost implications with subsequent delays in patient access to medical products [4, 15]. The lack of regulatory policy harmonisation between countries is among the reasons for backlogs and NMRA staff duplicating efforts [4, 15]. Furthermore, in light of the globalisation of pharmaceutical manufacturing, it is very difficult for an NMRA to effectively regulate all medical products on its market [11, 23, 24]. In the present context of linked supply chains, one country is increasingly dependent on the quality and safety systems that are in place in another country [11, 23].

In some African countries, the availability of essential medical products is potentially delayed by disparities in legal provisions of key regulatory functions resulting in the need for regulatory convergence towards a common framework [20]. Therefore, in January 2016 the African Union (AU) Model Law on Medical Products Regulation was officially endorsed. This model law was developed and promoted by the New Partnership for Africa’s Development (NEPAD) Agency, now referred to as the African Union Development Agency NEPAD (AUDA-NEPAD), to provide the legislative framework for good medicine regulation at a national level. In addition, the model law provides the national legislative framework for harmonisation at the regional and sub-regional level, as well as to increase efficiencies in regional, sub-regional and national procedures. It is legislation meant to be domesticated and implemented by AU Member States and RECs for regulatory systems harmonisation, to facilitate collaboration across countries, and to ensure that in countries involved in research and development medical products that hold promise are developed, tested, and scaled up for the improvement of health impact [4, 9, 14, 15, 25].

In response to national medicines regulatory challenges, the African Medicines Regulatory Harmonisation (AMRH) initiative was formalised in 2009 [5, 20, 25]. Its main aim is to create more effective, efficient and transparent regulatory mechanisms in various African markets through collaborative regional mechanisms that, among others, achieve faster medical product approvals [18, 20, 25, 26]. The AMRH initiative intends to expand its scope of work gradually, commencing with generic medicine registration and moving towards oversight of vaccine clinical trials, pharmacovigilance, and the registration of New Chemical Entities (NCEs), medical devices and diagnostics [16, 19, 20]. The AMRH initiative also proposes to serve as the foundation for the establishment of the African Medicines Agency (AMA) [1, 7, 11, 19, 27–29].

This review maps the key entities, milestones, implementation targets and anticipated challenges related to the AU Model Law’s domestication and the AMA’s establishment. It also highlights the benefits and challenges of medicines regulatory harmonisation. We find that implementation targets for the AU Model Law have not been fully met, and the AMA treaty has not been ratified by the minimum required number of countries for its establishment. In spite of the challenges, the AU Model Law and the AMA hold promise to address gaps and inconsistencies in national regulatory legislation as well as ensure effective medicines regulation by galvanising technical support, regulatory expertise and resources at a continental level. Furthermore, this review provides recommendations for future research.

## Regulatory systems and maturity level in Africa

All African countries, with the exception of Sahrawi Republic, have an NMRA or an administrative unit conducting some or all expected NMRA functions [20]. However, only 7% have moderately developed capacity and over 90% have minimal to no capacity [20]. In addition, African NMRAs have varying corporate profiles with some lawfully established as body corporate, whereas others operate as departments or units under their respective Ministry of Health [20, 26, 30, 31]. The NMRAs also have variable functionalities and they are at different growth, expertise and maturity levels [20]. The ‘maturity level’ concept is incorporated in the Global Benchmarking Tool (GBT) used by WHO to objectively evaluate regulatory systems [32]. The GBT allows WHO and NMRAs to assess the regulatory system’s overall maturity on a scale of 1 (the existence of some regulatory system elements) to 4 (operation is at an advanced performance level and there is continuous improvement) [32]. Africa has no NMRA operating at maturity level 4. However, the NMRAs of Ghana and Tanzania operate at maturity level 3 [33]. All NMRAs on the continent eventually report to their Ministry of Health as the Minister has overall responsibility [20]. Regardless of the differences in organisational structures and remits, African NMRAs have for many years managed a diverse range of responsibilities and issues affecting medical product regulation, most often with limited resources [17]. Their main focus has been to ensure that the populations that they serve have access to a range of affordable essential medical products, which are usually generic medical products [17]. Therefore, NMRAs may have experience in the management of generics and have limited experience in NCE assessment, approval and registration [17].

Most African countries have medicines policies which support medical product regulation. A situational analysis in WHO-AfRO revealed that 40 of the 46 countries surveyed have medical product legislation, although only 15% of the NMRAs have a legal mandate to perform all five critical regulatory functions: marketing authorisation, pharmacovigilance, post-market surveillance, quality control, and clinical trials oversight [20]. Additionally, the regulatory oversight level has wide divergence with few member states having NMRAs that are robust and functional whereas other member states have regulatory systems that are virtually non-existent [5, 7, 13–15, 20, 21]. The regulatory approaches and needs differ as a result of resource base, general development levels, economic development, infrastructure, prevailing healthcare systems, research capacity and political commitment [7, 8, 18]. Although WHO recommends NMRAs to regulate all types of medicines, of 26 African NMRAs included in a study, 65% have a mandate to control veterinary medicines, 69% have provisions for traditional/herbal medicine regulation, and 65% regulate a broad range of products including foods, pesticides, bottled water, cosmetics and/or animal food supplements [20]. There is also a need for more robust pharmacovigilance systems as only 72% of countries in Sub-Saharan Africa (SSA) have quality control laboratories albeit at different developmental levels, and 63% are engaged in market surveillance [20].

Over the past decade, the regulatory landscape in Africa has undergone considerable transformation [20]. However, there are existing and emerging medicine registration issues in Africa which include the regulation of biosimilars and vaccines, advancements in medical products, clinical trial regulation and the establishment of clinical trial registries, blood and blood product regulation, and regulation of medical devices, especially diagnostic agents [7, 17, 23]. There are also reported delays of 4–7 years between first regulatory submission to a well-resourced NMRA and final approval in SSA [19, 20]. Some of the barriers that cause these delays are lengthy processes for medical products registration, general resource constraints, and failure to leverage regulatory review activities that have been carried out by stringent regulatory authorities [19]. Furthermore, the challenge of regulating medical products is exacerbated by the continent having more than 70% of medical products that are consumed being imported, which fuels illegal drug transactions as well as contributes to the consumption and circulation of substandard and falsified (SF) medical products [19].

## The African Union Model Law on Medical Products Regulation

With the aim of ensuring the promotion of innovation and access to new health technologies, the AUDA-NEPAD and key stakeholders developed the AU Model Law on Medical Products Regulation, hereafter referred to as the AU Model Law [14, 15]. The goal of this non-prescriptive model legislation is to streamline regulatory systems and facilitate the overall regional harmonisation process [4, 9, 14, 15, 19–21, 25]. The history of the AU Model Law is that the draft law was developed through the AMRH initiative platform and endorsed by the Pan African Parliament Committee on Health, Labour and Social Affairs [7, 21]. In November 2015, the AU technical committee on Justice and Legal Affairs approved the AU Model Law which is available for use as a starting point for the establishment of regulatory bodies and providing support for legislation in AU Member States [9, 21]. In January 2016, the AU Model Law was then endorsed at the AU Summit in Addis Ababa, Ethiopia by the AU Heads of State and Government [10, 14, 20, 34]. Following endorsement, next steps taken were to engage with RECs, regional organisations (ROs), and member states to update and enact regional legal frameworks and national laws on the regulation of medical products [14, 20, 34].

**Fig. 1:**
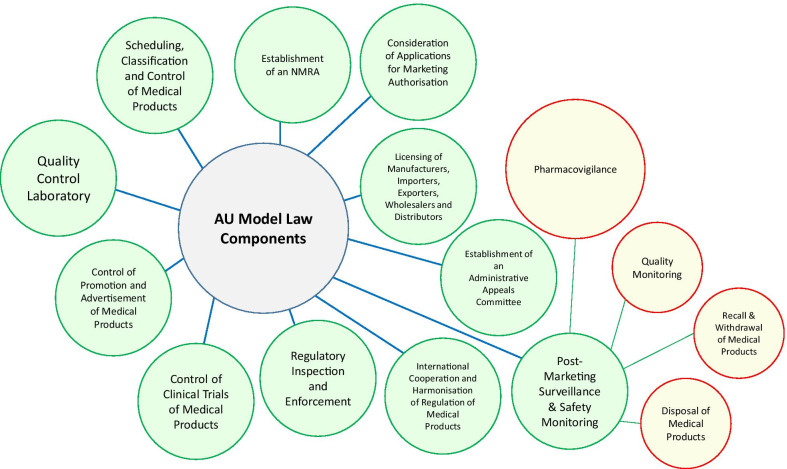
Key components of the AU Model Law

The objective of the AU Model Law is to increase collaboration across countries and to ensure that medical products that hold promise are developed, tested, and scaled up for the improvement of health impact [4, 9, 14, 15, 25]. The AU Model Law also supports the AU’s desire to promote local pharmaceutical production, with the goal of public health protection and economic growth [4], as well as supports continental efforts to advocate for, and catalyse access to novel medical products for patients in need [14, 19, 20]. Through the AU Model Law domestication process, a country can adapt the AU Model Law so that it is consistent with its constitutional principles and legal system, as well as amend or repeal any inconsistent national laws in force [15, 19, 25, 35]. The AU Model Law is available in four different languages, i.e. English, French, Portuguese, and Arabic, and is intended to serve as a template for AU Member States to adopt best practices in medical products regulation into their national laws [14, 19, 20]. The key components of the AU Model Law are presented in Fig. 1.

For African countries without internationally comparable laws, the implementation of the AU Model Law is expected to have an impact on the national regulatory system and the implementation benefits can be seen at the technical level as well as at the more general health systems level [34]. Regarding the broader health system, the AU Model Law implementation benefits include: (i) having national laws that are up to international standards, allowing the respective governments to catalyse access to innovative and lifesaving medical products for citizens [15, 34]; (ii) supporting access to health by ensuring medical product availability; (iii) supporting, in the respective country, effective market control for medical products that are in circulation; and (iv) having legal provisions at the national level that allow regional harmonisation and international collaboration [34]. Through AU Model Law implementation, AU Member States strengthen national and regional regulatory systems as well as reduce SF medical product prevalence through the enforcement of a provision for SF medical product prohibition [19].

## Country-level adoption of the AU Model Law

At a national and regional level, implementation targets related to the AU Model Law are to have at least three regions adopting regional policies and legal frameworks for medical product regulation by 2020 [34], and at least 25 countries domesticating the AU Model Law by 2020 [14, 19, 34]. Currently, 17 countries have adopted or adapted the AU Model Law [36] and they include Burkina Faso, Burundi, Ivory Coast, Lesotho, Mozambique, Namibia, Rwanda, Seychelles, The Gambia, the Kingdom of Eswatini, United Republic of Tanzania (Zanzibar), and Zimbabwe. These countries offer lessons and best practices that can be emulated when revising national medicines regulatory systems using the AU Model Law as the reference document [10, 14, 19, 20, 34]. They offer examples of domesticating and implementing a version of the AU Model Law that best responds to a country’s respective needs in order to set up a streamlined regulatory system that ensures that medical products meet international standards of quality, safety and efficacy [34]. Member States are called to use the AU Model Law and bring the commitments made by their respective governments at the continental level to fruition [34].

For the successful implementation of the AU Model Law, RECs, ROs and member states are encouraged to carry out a combination of preliminary situational and needs assessments on the existent medicines regulatory system, including existing frameworks, in individual countries using the AU Model Law as the benchmark [14, 34]. Based on the gaps that are identified, a roadmap should be developed for AU Model Law implementation that clearly outlines the action plan to address the gaps, and if feasible, RECs should harmonise regulatory requirements for their member states [14, 34]. We note that there is a paucity of information on how many countries have laws deemed to be sufficient/comprehensive and already satisfy the AU Model Law requirements. Therefore, research needs to be carried out to address the foregoing as well as to assess countries pre- and post-AU Model Law implementation. The AU Model Law and AMRH initiative efforts are intended to support countries to rectify some of the regulatory challenges that they have been grappling with for many years [15]. The long term goal of the AMRH initiative is to establish the African Medicines Agency, which will have the mandate of overseeing the registration of specific medical products and coordinating regional harmonisation systems in Africa [14, 37]. Therefore, the AU Model Law’s development is interpreted within the context of these overarching efforts towards regulatory harmonisation in Africa [14].

## Establishing the African Medicines Agency

**Fig. 2:**
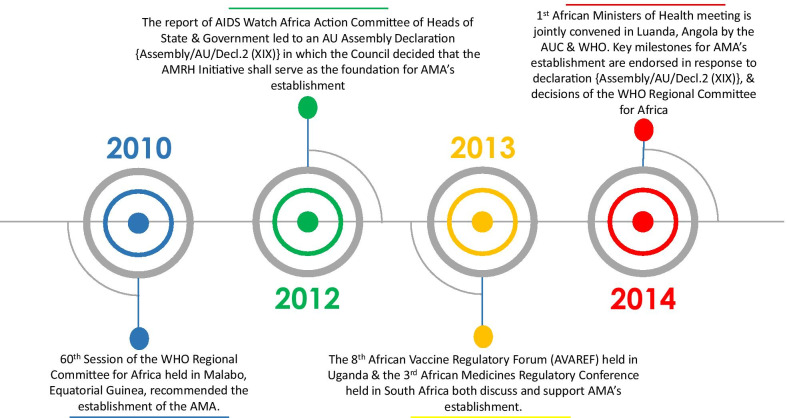
The historical context of the African Medicines Agency

In Africa, regulatory systems in some countries are better than in others [14, 30, 31]. These regulatory capacity disparities are considered a basis for the establishment of a continental regulatory system [30, 31]. To effectively address some of the challenges that are being faced by African countries, AU Member States are establishing the AMA. These challenges include implementing agreed procedures and processes, coordinating regulatory practices across sub-regions, priority setting for medical products against select diseases, pharmaceutical manufacturing promotion and optimally using the NMRA’s limited resources [11, 30, 31]. The establishment of AMA is based on AU Executive Council Decision EX.CL/Dec.857 (XXVI) of January 2015 [19, 38]. In addition, Africa has several donors and networks for regulators which aim to enhance the availability of medical products and serve as opportunities for improving regulatory convergence at a continental level. Therefore, these donors and networks could potentially benefit from the regulatory oversight which the AMA’s establishment would provide [11, 30, 31]. Moreover, the alignment of regulatory systems strengthening efforts, harmonisation initiatives and advocating for AMA’s establishment are important for optimising pharmaceutical markets as well as ensuring the sustainable supply of medical products for priority and neglected diseases [19]. Figure 2 shows the historical context of the African Medicines Agency.

### The African Medicines Agency Treaty

**Fig. 3:**
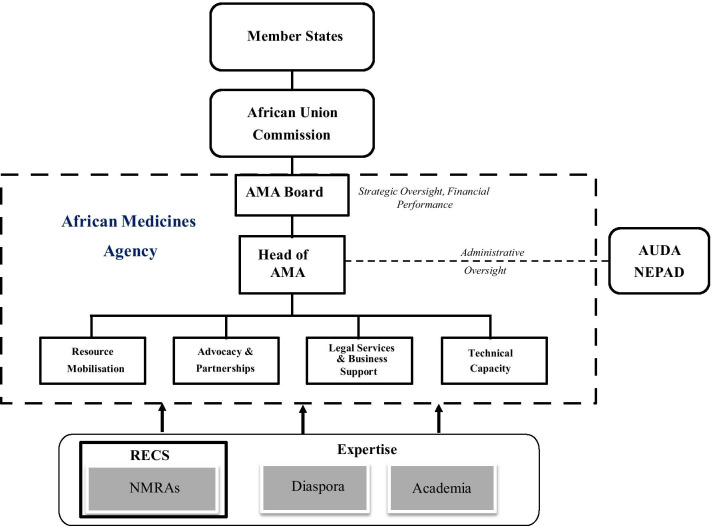
The proposed structure of the AMA. The proposed governance structure of AMA includes Member States, a Board with strong African government and technical representation, key stakeholders and a Secretariat led by the Head of AMA. AMA’s Board will be responsible for strategic oversight and direction, financial performance and accounts to the Member States through the AUC. The Secretariat will be responsible for operational performance, strategy/business plan implementation, as well as coordination/facilitation of medicines regulatory activities and harmonisation. The AMA’s structure intends to ensure the maintenance of a lean staff and the use of both internal staff and experts from participating NMRAs. The role of key staff will therefore be the coordination of AMA’s activities. The European Medicines Agency (EMA) and WHO PQTm have used similar approaches. The Head of AMA will be supported by a resource mobilisation team, an advocacy and partnership team, a legal services team and a technical capacity team [11]

The AMA is to be established through a treaty which takes into consideration key AU decisions, declarations and policy frameworks including the 55th Decision of the AU {Assembly/AU/Dec.55(IV)} taken during the 2005 Abuja Summit and the 19th Ordinary Session Decision of the Assembly {Assembly AU/Dec.442(XIX)} [1, 4]. On 11 February 2019, the AU Assembly, during their 32nd Ordinary Session in Addis Ababa, Ethiopia, adopted the treaty for the establishment of the AMA [38–40]. The treaty was then unanimously adopted by the African Ministers of Health gathered at the 71st World Health Assembly in Geneva [24, 28]. The AMA was expected to be launched in 2018 [25, 30], with efforts being made to ensure that the agency capitalises on already existent mechanisms, experiences and technologies to work in an effective manner towards the accomplishment of its objectives [30]. It has been estimated that in the first 5 years, a total of US$100 million will be required to fund the AMA with portions of this amount funding human resource costs, infrastructure and operational costs [31]. The AU Member States have also agreed to provide contributions in kind to the AMA by way of dedicating part of the time of their NMRA staff for the work of the Agency [11, 30, 31, 38]. This is to ensure that the AMA has a small critical mass of competent staff to enable the work of the experts, and that of their respective committees [11, 30]. The proposed structure of the AMA is illustrated in Fig. 3.

In the context of moving towards AMA’s establishment, the AMA treaty must be signed and then ratified. Ratification refers to the national procedure where the member state puts in place a law that allows for the implementation of the AMA treaty [41]. African health leaders are currently adopting the treaty and on 12 June 2019, Rwanda became the first AU Member State to sign the treaty [1, 28, 40]. As of September 2020, there are 17 AU Member States that have signed the treaty: Algeria, Benin, Burkina Faso, Chad, Ghana, Guinea, Gabon, Madagascar, Mali, Morocco, Niger, Rwanda, Sahrawi Republic, Senegal, Sierra Leone, Seychelles, and Tunisia. Only Burkina Faso, Ghana, Mali, Rwanda and Seychelles have ratified the treaty [42]. Literature shows that the lack of political commitment within countries is one factor that could impede the implementation of regional or continental decisions. Misconceptions, particularly at lower levels of integration, should therefore be resolved along with any differences in policy [11, 13]. Other known factors exist which may further influence the establishment and envisaged success of the AMA such as:

1. Language barriers: the AU has at least six official languages with some RECs having more than two official languages;
2. The creation of the African Continental Free Trade Area (AfCFTA): progress in this regard will have an impact on AMA’s progress as the agency’s activities will be conducted within the context of regional/continental integration;
3. The functionality of Regional Centres of Regulatory Excellence (RCOREs): regulatory capacity at NMRAs can be built through the optimum use of the established RCOREs;
4. Political and policy leadership to support efforts in harmonisation at the AU and RECs; and
5. Sustainable financing mechanisms [11].

### The value proposition of the African Medicines Agency

Intended to be an organ of the AU that is legally mandated by member states, AMA aims to provide a platform for the coordination and strengthening of ongoing medicines regulatory harmonisation initiatives across the continent [1, 11, 24, 30, 31, 43]. It plans to ensure optimal use of scarce resources by pooling expertise, capacities and strengthening existing networks. The AMA is also intended to offer guidance, in addition to complementing and enhancing the harmonisation efforts of RECs. This will theoretically contribute to enhanced accessibility of quality-assured and affordable medical products [1, 11, 24, 27, 30, 31, 39, 43, 44].

AU Member States have recommended that the establishment of AMA be done in a stepwise approach that involves the AUC and RECs [30, 31]. Under the leadership of the AMA, efforts in regulatory systems strengthening and harmonisation initiatives can be better coordinated. This may result in improved sovereign control and medical products regulation that allows AU Member States to provide protection for public health more efficiently and effectively, particularly against risks associated with SF medical product use [11, 27, 39, 40]. Furthermore, the AMA proposes to enable expedited approvals for medical products that meet the health needs of Africans, particularly for conditions that affect Africa disproportionately, while also fostering the competitiveness of locally manufactured medical products [11, 27]. Ultimately, instead of having 54 NMRAs on the African continent, each with its own regulatory requirements, the AMA intends, among other goals, to result in streamlined regulatory processes in order to enable the timely evaluation and subsequent registration of medical products [11].

#### Vision and mission of the AMA

The AMA’s vision is to ensure that all Africans have access to quality-assured, safe, efficacious and affordable medical products, that meet internationally recognised standards, for priority diseases or conditions [11, 30, 31, 39, 40]. At the continental level, AMA’s mission is:

1. To coordinate national and sub-regional medicines regulatory systems;
2. To conduct regulatory oversight of selected medical products including traditional medicines; and
3. To promote cooperation, harmonisation and the mutual recognition of regulatory decisions [11, 20, 24, 30, 31, 39, 40, 44].

AMA proposes to work collaboratively with NMRAs, provide technical guidance, reduce duplicative efforts, and ensure cost-effective use of limited resources [20]. In order to achieve its mandate, the AMA also intends to work with technical partners such as WHO, the EMA and US Food and Drug Administration for relevance and participation on normative standards, technical cooperation and capacity building [11]. In addition, improved access to quality-assured medical products may result from an enhanced regulatory environment created by AMA [30]. The AMA, serving as a reference centre that has a coordination and stewardship function for the regulatory activities of AU Member States, intends to perform the following as part of its core activities: (i) marketing authorisation; (ii) joint assessments and GMP Inspections; (iii) market surveillance; (iv) safety monitoring; (v) oversight of clinical trials; and (vi) coordination of quality control laboratory services [11, 27, 30, 31].

#### Medicines assessment

There is a dearth of information on the extent of the quality and safety of medical products in African countries as a result of inadequate regulatory and post-marketing surveillance systems [11]. Compared to medicines, the situation for medical devices and in vitro diagnostics is postulated to be worse due to the relatively limited capacity to regulate these products [11]. Therefore, for functions such as GMP inspections of foreign manufacturers, reviewing complex medical products and multi-country clinical trials, AMA and regional agencies can optimise available resources within RECs by harmonising technical requirements and work sharing activities, as well as coordinating technical support for AU Member States [11, 38, 39]. It is worth noting that AMA will not replace NMRAs or the sub-regional medicines regulatory authorities which will be established by RECs [11, 19, 38, 39]. Instead, the AMA desires to complement the efforts of NMRAs, RECs and ROs intending to create a conducive environment for the pharmaceutical industry to develop through enhanced coordination of the various stakeholders involved in African regulatory harmonisation initiatives [19]. NMRAs will still assess the majority of medical products, have their regulatory decision making roles and put in place market controls for their specific territories [11, 19]. As the AU does not have sweeping legal powers over the national jurisdictions of member states, decisions made at the continental level are not legally enforceable in AU Member States [11]. Table 1 shows the level of implementation of regulatory functions at the NMRA, regional and AMA level.

**Table 1:** Level of implementation of regulatory functions at national, regional and continental level [11]

Regulatory function	NMRA	Regional harmonisation	AMA
Registration of medical products	X	X^a^	NA
GMP inspection of manufacturers	X	X^b^	X^c^
Inspection of supply chain (importers, wholesalers, retailers)	X	–	–
Post-marketing surveillance	X	X^d^	X^d^
Pharmacovigilance	X	–	–
Regulation of clinical trials	X	X^e^	X^f^
Quality control	X	–	–
Medicine information	X	–	–

^a^ In some RECs, centralised registration may not be feasible as it is dependent on specific regional contexts. In addition, centralised registration will only be for selected products for which centralised registrations would offer a comparative advantage

^b^ The majority of NMRAs do not have the resource capacity to perform GMP inspections. Therefore, this function can ideally be done at both the national and regional level, though NMRAs have the final approval

^c^ In African countries, GMP inspections of API manufacturers, biologics and vaccines is virtually non-existent. Therefore, this function can ideally be coordinated and conducted at the continental level, though NMRAs have the final approval

^d^ Regional agencies and the AMA have the role of coordinating and facilitating information exchange at national, regional and continental level, particularly for SF medical products

^e^ Review and/or coordination of regulatory oversight of multi-country clinical trials

^f^ Regulatory guidance and/or coordination of regulatory oversight of clinical trials for investigative and innovative therapies (e.g. for pandemics such as Ebola and COVID-19)

#### Developing regulatory science specialists

Building on the experiences and strengths of the RCORE model, AMA intends to be an agency focused on developing regulatory science specialists [11, 24]. An RCORE is an institution, or partnership of institutions, with specific expertise in regulatory science as well as proven capacity and capabilities in the training or delivery of services in at least one of the categories of regulatory and managerial functions that have been identified [19, 45]. Since 2014, the AMRH initiative has spearheaded the designation of 11 RCOREs that specialise in 8 regulatory functions, strengthening the development of regulatory capacity by leveraging existing academic, scientific/research and regulatory institutions [10, 19, 20, 25, 45]. These RCOREs are specialised in pharmacovigilance, training in core regulatory functions, quality assurance and quality control, medicines registration and evaluation, licensing of the manufacture, import, export, and distribution of medical products, inspection and surveillance, and clinical trials oversight [19, 25, 45].

AMA intends to offer regulatory guidance on particular issues that are problematic for which technical capacity and expertise are limited at the national or regional level [11, 24, 39, 44]. In addition, by providing recommendations that AU Member States can use as a basis for their own regulatory decision making, AMA potentially builds on the strengthened capacity of medical product/health technology regulation in Africa [1, 38]. Some countries in Africa have not fully exploited the Trade-Related Aspects of Intellectual Property Rights (TRIPS) flexibilities and this has been attributed to the technocrats who are tasked with dealing with Intellectual Property Rights (IPR) and access to medicines having generally limited knowledge on the subject area. There are also capacity constraints that include weak legal/regulatory frameworks and weak administrative capacity [13]. Therefore, having harmonised medicine registrations can assist the AU, through AMA, to effectively use TRIPS flexibilities for the production and import of generic medical products that are protected by patents in one or more African countries [43].

## Medicines regulatory harmonisation in Africa

### The role of regional economic communities in regulatory harmonisation

Regulatory harmonisation refers to the process of NMRAs aligning technical requirements for the development and marketing of medical products [46]. In Africa, there are 8 RECs: Arab Maghreb Union (UMA); Common Market for Eastern and Southern Africa (COMESA); Community of Sahel-Saharan States (CEN-SAD); East African Community (EAC); Economic Community of Central African States (ECCAS); Economic Community of West African States (ECOWAS); Intergovernmental Authority on Development (IGAD); and the Southern African Development Community (SADC) [47]. Within the framework of the Pharmaceutical Manufacturing Plan for Africa (PMPA), the AMRH initiative has been implemented, in collaboration with WHO and partners, with the intention of supporting the strengthening of medical product regulatory systems in these RECs and member states [7, 10, 11, 16, 38, 43]. The partnership has resulted in RECs and regional health organisations, which have been supported to serve as regional information sharing platforms, benefitting from regulatory requirements, standards, systems, legislation and practices that are harmonised [10, 11, 26]. The intention of the work done by RECs is to be a stepping stone for the harmonisation of activities in Africa [11].

Launched on 30 March 2012 [21], the EAC medicines regulatory harmonisation (MRH) project was the first successful regional group of the AMRH initiative [9] and it signalled the beginning of the implementation phase of the initiative across Africa [5, 19]. In 2015, the SADC MRH project was launched and it absorbed the ZaZiBoNa collaborative medicines registration initiative [19, 36]. In the same year, the MRH programme for the West African region was launched in Accra, Ghana, focusing on the development of national and regional GMP roadmaps [19, 25, 29]. In addition, the Organization of Coordination for the Fight Against Endemic Diseases in Central Africa (OCEAC) became involved in the AMRH initiative in July 2015 [29]. In April 2016, the IGAD member states signed the Khartoum Declaration to Call for Action towards medicines regulatory collaboration and harmonisation programme implementation [19, 25]. Through the RECs, the AMRH initiative has established a regional platform for medical products and health technologies’ regulation which can be utilised for the building of trust, confidence, ownership as well as alignment especially for countries that are in the process of building medicines regulatory systems [10, 11]. The RECs have also supported medicines registration harmonisation by creating common pharmaceutical policies and operational plans backed by high-level political commitments and mandates [16, 18].

### Challenges encountered in medicines regulatory harmonisation

Regulatory harmonisation in Africa is a challenge as a result of the wide array of regulatory environments and capacities [13]. Countries have different sovereign approaches to their legal and regulatory frameworks based on their own sociocultural values, as well as historical and political landscapes [48]. This is one of the aspects that the AU Model Law can assist in after it has been adopted and implemented by the countries. In addition, regulatory divergence across borders can be a result of differences in the degree of acceptable risks and benefits, disease burden, vulnerable populations, and costs [48]. Harmonisation is also made more challenging by gaps in the development of a unified regulatory science body and the availability of a competent regulatory workforce [48]. In order for medical products regulation to be effective and yield the envisaged benefits, all aspects of regulation must be addressed [8], and regulators should adapt medicines regulatory harmonisation activities based on local circumstances [9]. It is also important for medicines regulatory harmonisation to take into consideration the different commercial, regulatory and healthcare interests [7].

Drawing lessons from the SADC region, potential barriers to harmonisation are related to differences in organisational structures of the NMRAs, legislative and regulatory provisions, and guidance documents [9]. Another regulatory harmonisation challenge is having differences in risk–benefit decisions and interpretation of legislation used by NMRAs for regulatory and product approvals [9]. For instance, the AU Model Law is being interpreted, domesticated and implemented differently according to the local context and needs [9]. Even in situations where the same legal and scientific frameworks are used, NMRAs are going to have different priorities in terms of risks and benefits during medical product regulation [9]. Moreover, the fact that the final decision on marketing authorisation, after completion of the product’s technical assessment, is still the prerogative of each NMRA is in itself a harmonisation challenge [9, 36]. Other challenges encountered by countries in the SADC region’s ZaZiBoNa initiative include long registration review times owing to increased applications, backlogs, inadequate numbers of assessors, a lack of competency in the assessment of certain products such as biologicals and biosimilars, as well as a lack of clarity in country-level ZaZiBoNa processes [36].

Like the SADC region, the EAC region has no license that is valid for use in all its member states and the different NMRAs have the sole responsibility of granting marketing authorisations [5]. The EAC member states have also implemented higher levels of quality control in their harmonisation initiatives [5]. Their joint assessments require bioequivalence studies, whereas applications for marketing authorisation through national marketing authorisation pathways tend to waive such requirements [5]. In addition, some EAC NMRAs refuse to accept a joint decision [5], and this might be due to the economic model of NMRAs [9]. NMRAs obtain a significant portion of their funds from conducting GMP assessments, dossier reviews and other regulatory functions [5]. Through harmonisation and central registrations, this source of income would be lost. Regardless of funding sources, a recurring theme in regulatory harmonisation is that sustainable financing is a major barrier [9].

Garnering support for regulatory harmonisation is riddled with challenges [49], and although these challenges exist, regional harmonisation is possible and is occurring. In the EAC and SADC regions, NMRAs have jointly assessed dossiers through collaborative regulatory procedures (CRP) [20]. The NMRAs in these RECs have also conducted joint GMP inspections to enable faster product marketing authorisation [20]. In the EAC region, CRP work has resulted in a 40–60% reduction in medicine approval timelines for a number of branded medicines [20]. Additionally, the SADC region’s ZaZiBoNa initiative has demonstrated that work sharing can successfully occur due to leadership commitment, consistency and ownership [36]. Through ZaZiBoNa: medicine registration has become faster than it would if NMRAs worked independently, maximum output has resulted from sharing limited resources, and NMRAs have benefitted from capacity building [36]. Therefore, the AMA can capitalise on these already existing harmonisation initiatives to work in an effective manner towards the accomplishment of its objectives [30]. Moreover, the AMA can potentially overcome these harmonisation challenges and facilitate harmonisation by galvanising technical support, regulatory expertise and resources at a scale that neither the national nor regional initiatives can match [11].

## Outlook and recommendations for the future

This article reviewed the AU Model Law and found that its aims include to facilitate the overall regional harmonisation process and increase collaboration across countries. In addition, the article reviewed the AMA, which is being established by treaty to effectively address some of the challenges that are being faced by African countries, and reports that the AMRH initiative serves as the foundation for its establishment. This review also shows that implementation targets for the AU Model Law have not been fully met, and the AMA treaty has not been ratified by the minimum required number of countries for its establishment. The noted challenges related to regulatory harmonisation include countries having different sovereign approaches to their legal and regulatory frameworks, regulatory divergence across borders, inadequate financial resources, gaps in the development of a unified regulatory science body and the lack of a competent regulatory workforce. Against this backdrop, we recommend the following:

i. An assessment of the current status of implementation of the AU Model Law by AU Member States as it may provide a foundation for identifying the existent gaps and opportunities for improving medical product/health technology regulation, public health protection and promotion, and pharmaceutical industry advancement on the continent.
ii. An examination of the enabling factors and challenges encountered in domesticating and implementing the AU Model Law in AU Member States.
iii. An analysis of the enabling factors and barriers encountered by AU Member States in signing and/or ratifying the treaty for AMA’s establishment, with lessons also being drawn from Burkina Faso, Ghana, Mali, Rwanda and Seychelles’ experiences of treaty ratification.
iv. A comparative study between the AMA initiative and other continental initiatives in order to draw lessons from their implementation and find areas of applicability to Africa.
v. An investigation of African NMRAs’ expectations of the AMA, perceptions of their contributions to/in the AMA, and the perceived benefits of the AMA to their respective countries.

## Conclusions

Regulatory harmonisation offers several benefits to the various pharmaceutical stakeholders in Africa, including industry and patients. However, the effective regulation of medicines that guarantees public health protection is a complex undertaking, needing the application of robust medical, scientific and technical competency within the context of an appropriate legal framework. Therefore, the AU Model Law and the AMA hold promise to address gaps and inconsistencies in national regulatory legislation as well as to ensure effective medicines regulation by galvanising technical support, regulatory expertise and resources at a continental level.

## Abbreviations

AMA: African Medicines Agency
AMRH: African Medicines Regulatory Harmonisation
AU: African Union
AUDA-NEPAD: African Union Development Agency New Partnership for Africa’s Development
CEN-SAD: Community of Sahel-Saharan States
COMESA: Common Market for Eastern and Southern Africa
CRP: Collaborative regulatory procedures
EAC: East African Community
ECCAS: Economic Community of Central African States
ECOWAS: Economic Community of West African States
EMA: European Medicines Agency
GBT: Global Benchmarking Tool
GMP: Good Manufacturing Practice
IGAD: Intergovernmental Authority on Development
IPR: Intellectual Property Rights
MA: Marketing authorisation
MRH: Medicines regulatory harmonisation
NCE: New chemical entity
NEPAD: New Partnership for Africa’s Development
NMRA: National Medicines Regulatory Authority
OCEAC: Organization of Coordination for the Fight Against Endemic Diseases in Central Africa
PMPA: Pharmaceutical Manufacturing Plan for Africa
RCOREs: Regional Centres of Regulatory Excellence
REC: Regional Economic Community
SADC: Southern African Development Community
SF: Substandard and falsified
SSA: Sub-Saharan Africa
TRIPS: Trade-Related Aspects of Intellectual Property Rights
UMA: Arab Maghreb Union
USFDA: US Food and Drug Administration
WHO: World Health Organization
